# Nicotine patches and quitline counseling to help hospitalized smokers stay quit: study protocol for a randomized controlled trial

**DOI:** 10.1186/1745-6215-13-128

**Published:** 2012-08-01

**Authors:** Sharon Cummins, Shu-Hong Zhu, Anthony Gamst, Carrie Kirby, Kendra Brandstein, Hillary Klonoff-Cohen, Edward Chaplin, Timothy Morris, Gregory Seymann, Joshua Lee

**Affiliations:** 1 Department of Family and Preventive Medicine, University of California, 9500 Gilman Drive MC 0905, La Jolla, San Diego, CA 92093-0905, USA; 2 Department of Family and Preventive Medicine, University of California, 9500 Gilman Drive MC 0717, La Jolla, San Diego, CA, 92093-0717, USA; 3 Community Benefit Services, Scripps Mercy Hospital Chula Vista Well-Being Center, 237 Church Ave, Chula Vista, CA, 91910, USA; 4 Department of Family and Preventive Medicine, University of California, 9500 Gilman Drive MC 0607, La Jolla, San Diego, CA, 92093-0607, USA; 5 Department of Quality Services and Improvement, Scripps Mercy Hospital, 4077 Fifth Ave, San Diego, CA, 92103, USA; 6 Department of Medicine, UCSD Medical Center - Hillcrest, 200 West Arbor Drive, MC8378, San Diego, CA, 92103-8378, USA; 7 Department of Medicine, UCSD Medical Center - Hillcrest, 200 West Arbor Drive, MC8485, San Diego, CA, 92103-8485, USA; 8 Department of Medicine, UCSD Medical Center - Hillcrest, 200 West Arbor Drive, MC8490, San Diego, CA, 92103-8490, USA

**Keywords:** Smoking, Cessation, Telephone counseling, Nicotine replacement therapy, Hospital

## Abstract

**Background:**

Hospitalized smokers often quit smoking, voluntarily or involuntarily; most relapse soon after discharge. Extended follow-up counseling can help prevent relapse. However, it is difficult for hospitals to provide follow-up and smokers rarely leave the hospital with quitting aids (for example, nicotine patches). This study aims to test a practical model in which hospitals work with a state cessation quitline. Hospital staff briefly intervene with smokers at bedside and refer them to the quitline. Depending on assigned condition, smokers may receive nicotine patches at discharge or extended quitline telephone counseling post-discharge. This project establishes a practical model that lends itself to broader dissemination, while testing the effectiveness of the interventions in a rigorous randomized trial.

**Methods/design:**

This randomized clinical trial (N = 1,640) tests the effect of two interventions on long-term quit rates of hospitalized smokers in a 2 x 2 factorial design. The interventions are (1) nicotine patches (eight-week, step down program) dispensed at discharge and (2) proactive telephone counseling provided by the state quitline after discharge. Subjects are randomly assigned into: usual care, nicotine patches, telephone counseling, or both patches and counseling. It is hypothesized that patches and counseling have independent effects and their combined effect is greater than either alone. The primary outcome measure is thirty-day abstinence at six months; a secondary outcome is biochemically validated smoking status. Cost-effectiveness analysis is conducted to compare each intervention condition (patch alone, counseling alone, and combined interventions) against the usual care condition. Further, this study examines whether smokers’ medical diagnosis is a moderator of treatment effect. Generalized linear (binomial) mixed models will be used to study the effect of treatment on abstinence rates. Clustering is accounted for with hospital-specific random effects.

**Discussion:**

If this model is effective, quitlines across the U.S. could work with interested hospitals to set up similar systems. Hospital accreditation standards related to tobacco cessation performance measures require follow-up after discharge and provide additional incentive for hospitals to work with quitlines. The ubiquity of quitlines, combined with the consistency of quitline counseling delivery as centralized state operations, make this partnership attractive.

**Trial registration:**

Smoking cessation in hospitalized smokers NCT01289275. Date of registration February 1, 2011; date of first patient August 3, 2011.

## Background

Smokers are more likely than nonsmokers to be hospitalized. Most smokers in the U.S. actually do quit smoking when hospitalized, however, the majority of them return to smoking soon after their discharge from the hospital [1-3]. Patients who continue to smoke after hospitalization are more likely to be re-hospitalized compared to those who quit and maintain abstinence [4,5]. Thus, helping hospitalized smokers quit smoking and remain abstinent after discharge will save lives and reduce costs in health care [6-11].

Research has found that brief counseling provided to smokers while they are hospitalized has limited effect on prolonged abstinence [6]. A 2007 Cochrane Review on studies of hospitalized smokers suggests that interventions need to last at least one month post-discharge to have a statistically detectable effect. This suggests that more intense intervention is needed to reduce relapse after discharge from the hospital. Hospitals are not set up to provide this level of follow-up care [12,13]. Quitlines have a robust infrastructure to deliver counseling services by telephone and are well suited to provide follow-up care. Meta-analyses have shown that telephone counseling is an efficacious intervention for smoking cessation [14]. Currently, publicly funded quitlines exist in every US state, collectively serving > 500,000 smokers each year [15]. Telephone counseling is convenient and can be delivered proactively, which can keep the rate of counseling high. The high rate of delivery of counseling is a critical factor for testing the intervention effect.

The nicotine patch is a widely used quitting aid. A 2008 Cochrane Review shows that the nicotine patch is an effective treatment for smoking cessation with an odds ratio of 1.66 [16]. However, it appears that nicotine replacement therapy (NRT) has little effect with hospitalized smokers [6]. The causes for the lack of NRT effect in this context are poorly understood. Moreover, not all hospitalized smokers use nicotine patches during their hospital stay. And fewer still leave the hospital with them, which may contribute to a quick relapse after discharge.

Telephone counseling is widely available and quitlines are well equipped to provide extended follow-up care. Nicotine patches are widely used but not systematically and proactively delivered to hospitalized smokers. Hospitals and quitlines can work collaboratively to intervene with this vulnerable population to help hospitalized smokers stay quit after discharge.

The current study is based on pilot work conducted by our research team at the California Smokers’ Helpline (CSH) in partnership with a local hospital (Scripps Mercy, San Diego). Using a two-group design (usual care (UC) versus nicotine patches at discharge plus proactive quitline counseling), 126 subjects were recruited, randomized, and evaluated at two months. The intervention group was over three times more likely to be abstinent at follow-up than the usual care (*P* < 0.01). Equally important for the current study is that the pilot program tested all the experimental procedures and showed the feasibility of recruitment, intervention, and evaluation [17].

The model of a quitline-hospital partnership is important because the new hospital accreditation requirements (Joint Commission) related to tobacco cessation performance measures include follow-up with hospitalized patients to assess smoking status [18]. Since hospitals may find it difficult to comply with the follow-up component, they may be less likely to select tobacco cessation as one of their four performance measures. Hospital staff are already pressed for time and finding time and funding for follow-up would be problematic. If hospitals do select tobacco cessation as one of their measures, there is a strong incentive for hospitals to work with partners like quitlines to both assess smoking status and provide follow-up counseling. The potential impact of the quitline-hospital partnership is not simply about dividing the work load between hospitals and quitlines, but also about follow-up counseling being more likely to occur and nicotine patches being more likely to be used, which would result in better outcomes for patients.

The specific aims of this study are:

1. To demonstrate the effects of two interventions, dispensing nicotine patches at discharge and providing proactive telephone counseling after discharge, on abstinence rates of hospitalized smokers, using a 2 x 2 factorial design.

2. To compare the cost-effectiveness of three intervention conditions: patches alone, counseling alone, and the combined interventions, against the usual care condition.

3. To examine if a patient’s medical diagnosis (for example, cardiopulmonary) is a moderating factor for intervention effects such that patients with certain diagnoses benefit more from the interventions than patients with other diagnoses.

4. To establish a practical model of a hospital-quitline partnership that can be adopted by other state quitlines and hospitals.

## Methods

### Design

This study uses a 2 x 2 (patch by counseling) factorial design. Hospitalized patients are recruited from two health-care systems in San Diego County. Subjects provide signed informed consent and are randomly assigned by the computer to one of four conditions: usual care, proactive quitline counseling, nicotine patches at discharge, or both, in a 2 x 2 factorial design with an equal number of subjects per cell. Randomization is stratified by hospital and cigarettes per day (six to ten or eleven plus) and uses blocks of eight to keep a balance of characteristics across the four conditions. Smoking status is evaluated at baseline, two and six months after discharge. Cotinine-validated smoking status is assessed on all subjects who report abstinence and a random sample (25%) of subjects who report continued smoking at six months.

### Setting

This study is conducted through two health-care systems, University of California, San Diego (UCSD) and Scripps, with a total of five hospitals. These hospitals have a combined licensed capacity of over 1,400 beds (Scripps has 873 and UCSD has 531) and are responsible for over 65,000 inpatient admissions each year. Estimates for smoking prevalence among these inpatients are 15 to 20%, providing a large pool from which to recruit. San Diego has a diverse population, including a large Latino population, which is reflected in the hospital population. Quitline counseling is provided by the California Smokers’ Helpline (CSH), the state quitline that is centrally operated from San Diego.

### Study population

Subjects will be 1,640 English- and Spanish-speaking adult smokers (18 years and over) hospitalized at one of the participating hospitals.

#### Eligibility criteria

Hospitalized smokers are eligible for inclusion if they are adults who smoked in the previous 30 days and are interested in staying quit (or planning to quit) upon discharge. Subjects must speak English or Spanish and provide sufficient contact information for intervention and evaluation (for example, name, address, phone number). They must provide signed informed consent and must receive physician approval for study participation.

Exclusion criteria include an anticipated hospital stay of less than twenty-four hours (shorter stays do not provide sufficient time for the assessment, consent, and intervention), inability to communicate or provide signed informed consent, or smoking fewer than six cigarettes per day. Pregnant smokers are not included in this study because there is still debate about the advisability of using nicotine patches during pregnancy [19,20].

### Sample size

This study uses a factorial design and was powered by marginal means (that is, the mean of the counseling factor averaged across levels of the nicotine patch factor) rather than the interaction. Sample size estimates were conducted using R [21]. Based on pilot work conducted in one of the hospitals with 120 patients, we assume the abstinence rates at six months will be 7% in usual care, 14% in proactive quitline counseling, 14% in the group receiving patches at discharge, and 21% in the group receiving both proactive counseling and patches [17]. Simulating binomial data from the assumed distribution, fitting generalized linear (binomial) models to the resulting data, and applying Hochberg’s (1988) [22] set-up procedure to the results, indicates that 298 subjects per cell is sufficient to have 80% power to see both main effects with the family-wise error rate controlled at the 0.05 level (two-sided). Assuming a variance inflation of 10% due to clustering and average follow-up rates of 80%, we need 410 subjects per cell - or 1,640 total - to have the same power for the mixed-effects complete-case analysis of the primary outcome with composite Type 1 error rate ≤ 0.05 (two-sided).

Hospital records suggest that about 30% of admitted smokers have cardiovascular or pulmonary diseases. This means, about 492 of the 1,640 subjects will be in this group, which allows for analysis of the moderating effects of diagnosis on interventions.

### Human subjects’ approval

This study is funded by the National Cancer Institute through a U01 mechanism. It has received ethics approval from the University of California San Diego Human Research Protection Program (HRPP) (Number 110410), the Scripps Hospital System Internal Review Board (IRB) (Number 11-5695) and the Consortium of Hospitals to Advance Research on Tobacco (CHART) Data and Safety Monitoring Board (DSMB). Information about study subjects is kept confidential and managed according to the requirements of the Health Insurance Portability and Accountability Act (HIPAA) [23].

### Procedures

#### Recruitment process

**Figure 1:**
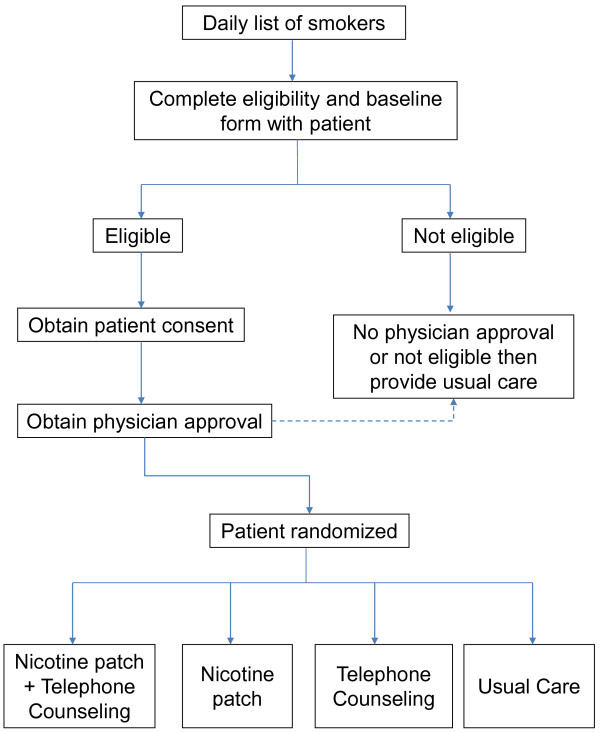
**Flow diagram of the CHART Trial at UCSD and Scripps Hospitals.** Eligible patients are stratified by hospital and cigarettes per day and randomized into one of four groups in a 2 x 2 factorial design.

This study is an effectiveness trial; therefore, every effort is made to embed the recruitment process into the current work flow of the hospital (Figure 1). The health-care systems have different procedures for addressing smoking with their patients. As a result, there are two recruitment/implementation models that differ in who has the responsibility for each task and in the degree to which they rely on automated processes through the electronic medical record (EMR). Both hospital systems routinely assess smoking status of all patients at the time of admission and record this information in the electronic medical record. As part of routine care, each day a list of recently admitted smokers is generated and designated hospital staff visit the smoker at bedside to provide a brief cessation intervention. In this study, the bedside visit is used to assess the patients’ eligibility for the study and to obtain signed informed consent. Patients who are not eligible for the study or not interested in participating receive the current standard of care, which is to provide the toll-free number for the California Smokers’ Helpline (1-800-NO-BUTTS).

In the Scripps hospitals, respiratory therapists (RT) are responsible for recruitment of subjects and for nicotine patch delivery at discharge. They are also responsible for contacting the physician to obtain approval for study participation. Although the medical record is flagged for patients who are randomly assigned to receive nicotine patches, the electronic medical record system is not used for communication between the RT and the physician or pharmacy.

At the UCSD hospitals, subjects are recruited into the study by research staff (aka research recruiter or RR) provided by the CSH research team. The electronic medical record (EMR) is used extensively to communicate between research and hospital staff with many of the processes automated. A daily list of recently admitted smokers is generated from the EMR and is viewable by RRs. The physician approval process is also embedded in the EMR system. In this hospital system, smokers are approved for participation in the study unless physicians indicate in the EMR that they should not participate.

#### Randomization

For this study, the CSH research team established and hosts a secure website that is used to randomize subjects into condition. The website complies with all HIPAA security guidelines for electronic protected health information (EPHI) [24]. All communication between hospitals and the California Smokers’ Helpline (CSH) is encrypted; identifying fields that are stored locally remain encrypted to ensure subject confidentiality. Staff responsible for recruitment and randomization enter the website through a password-protected web portal.

Recruitment staff (that is, RT and RR) complete the eligibility assessment and baseline measures, obtain signed informed consent from the smoker and approval from the physician. To complete the randomization procedure, staff use a web portal developed in-house to access the secure proprietary website. They enter the name, address, medical record number, phone number, cigarettes per day, and the anticipated discharge date of the smoker. The computer checks the name against a list of previously randomized subjects. If smokers are already in the study, a pop-up indicates their treatment condition and prevents re-randomization. If the smoker is not already in the study, the RT or RR confirms that the smoker is eligible, has provided signed informed consent, and that the physician has provided approval for study participation. The computer then randomly assigns the subject to condition. The condition is displayed to the RT or RR and all information is transmitted to CSH daily.

### Interventions

#### Telephone counseling

Telephone counseling is provided by the CSH, a state-funded service that is operated through the Moores Cancer Center at the University of California, San Diego. The quitline, which has been in operation since 1992, provides free cessation services to about 30,000 state residents each year from one location in San Diego, California.

The telephone counseling provided is, in effect, the standard service provided to any smoker who calls the state quitline for help except that a counselor initiates the first call. The telephone counseling uses a combination of social learning theory, cognitive-behavioral therapy and motivational interviewing to increase self-efficacy, resolve ambivalence, encourage new behaviors while changing self-defeating thoughts, and create a solid relapse prevention plan [25-27]. Subject information is uploaded into the CSH database daily and a list is generated each day of clients who need to be called. Counselors make at least 10 attempts to reach the client for the first counseling call. The initial counseling call is a 30 to 40 minute comprehensive session to discuss the client’s quitting experience during and after the hospital stay and to set up or solidify the quitting plan accordingly. After this call, counselors initiate up to eight follow-up calls to assess the client's progress and, as appropriate, discuss and normalize withdrawal symptoms, review proper use of pharmacotherapy, examine slip or relapse situations, revise the plan as needed and bolster self-efficacy and motivation. Follow-up sessions last about 10 to 15 minutes depending on the needs of the client.

The standard quitline telephone counseling has been described in great detail elsewhere [25] but, briefly, it has several distinguishing features including proactive counseling, a structured counseling protocol, and relapse-sensitive scheduling. The content of the counseling addresses both behavioral and cognitive issues the individual smoker faces in an attempt to quit. Calls are front-loaded when the probability of relapse is highest and spaced out as the client experiences success in quitting; such relapse-sensitive scheduling of calls has been shown effective in preventing relapse [28]. The telephone counseling is provided by a program with a mature infrastructure and rigorous quality assurance measures, which facilitates its implementation.

#### Nicotine patches

Providing nicotine patches can help with nicotine dependence. Perhaps more importantly, smokers who put on a nicotine patch at the time of discharge are making a statement of their intention to stay quit after leaving the hospital. Subjects randomized into the patch condition receive an eight-week supply of nicotine patches. At the Scripps hospitals, following randomization, the RT flags the medical record of subjects in the patch conditions and faxes a prescription to the pharmacy. At discharge, the RT retrieves the package of nicotine patches from the pharmacy and delivers it to the subject. At the UCSD hospitals, research staff query the list of subjects randomly assigned to receive patches and contact the pharmacy to prepare the package. Pharmacy staff deliver the nicotine patches to the subject at the time of discharge.

Patches are dosed according to how many cigarettes the subject smoked each day using a step-down regimen. Those who smoked six to ten cigarettes per day receive six weeks of 14 mg (Step 2) and two weeks of 7 mg (Step 3) patches. Those who smoked eleven or more cigarettes per day receive four weeks of 21 mg (Step 1), two weeks of 14 mg (Step 2), and two weeks of 7 mg (Step 3) patches. Subjects in the patch conditions who are not already wearing a patch are told to put on a patch before leaving the hospital.

#### Usual care

All subjects receive the hospital’s standard care (that is, usual care). This care typically includes a brief bedside intervention (<10 minutes) in which an RT or nurse encourages quitting and provides educational materials and the number for the state quitline. If it is not possible to make contact because the patient is sleeping or involved in a test or procedure, staff may leave the materials. Subjects might be provided with nicotine patches during their hospital stay, but this is not typical. There is no systematic assessment of their need for a quitting aid. Such decisions are made by the physician on a case by case basis.

### Data collection and measurements

It is important to gather data for the study in ways that are not overly burdensome for subjects or hospital staff. Therefore, we established some guiding principles for data collection. First, if data are already part of the electronic medical record, the information is obtained directly, rather than asking the subject to provide it again. This not only reduces the time for staff and subjects, it minimizes errors. Second, only essential items are collected at baseline. These items include those that: are needed to ensure eligibility for the study, allow delivery of the appropriate intervention, or are measures CHART has determined to be ‘core measures’ [29]. Third, sufficient data are collected at two- and six-month evaluations to allow analysis of multiple outcomes such as thirty-day abstinence, quit attempt rate, and prolonged abstinence. Fourth, process data regarding the counseling and patch intervention are collected throughout the study.

#### Data collection points

Initial data are collected from subjects while they are in the hospital (baseline). Subsequently there are evaluation calls at two and six months after baseline.

#### Measurements

All data that are considered ‘core measures’ to the CHART trials are collected [29], as well as additional data relevant to the interventions. The trial will be reported using the CONSORT guidelines for transparent reporting of trials [30].

### Baseline measures

Contact information (for example, name, address and phone), hospital site, medical record number, admission date and time, demographics (for example, age, sex, ethnicity/race, education, marital status, and type of insurance), and height and weight (used to determine BMI) are obtained from the medical record [29]. Other information that is required to assess eligibility and to measure the baseline is obtained directly from the subject. Subjects’ smoking status (that is, smoker versus nonsmoker) is based on the criterion of whether they report having smoked in the last 30 days. Other tobacco variables obtained at baseline include the number of days the subject smoked in the last 30, number of cigarettes smoked per day on average, use of any other form of tobacco, history of smoking since being admitted to the hospital, and confidence to stay quit after discharge. Subjects are only eligible for the study if they intend to stay quit (or plan to quit) after discharge. Because one intervention is the nicotine patch, subjects are asked about possible contraindications for nicotine patch use (that is, high blood pressure, heart attack, stroke, arrhythmia, angina and a history of severe allergic reaction to adhesive tape). Subjects who have a possible contraindication to using the nicotine patch are still eligible for the study, provided the physician approves.

### Discharge information

As part of the CHART consortium, this project gathers core measures about discharge from the medical record [29]. These measures include: discharge plan (home, skilled nursing facility, or other placement), discharge date and time (used to determine length of hospital stay and report timing of intervention activities), and information regarding diagnosis (that is, primary and secondary discharge diagnosis, diagnosis-related groups (DRG), and procedure codes).

### Evaluation measures

At two and six months, subjects are called by the UCSD evaluation team, a group that is independent of the quitline counseling staff. Up to 30 attempts are made to reach subjects before being coded as a ‘no contact’. Two weeks prior to the evaluation, a letter is sent to remind subjects of the research and to encourage their participation in the evaluation. Each letter includes a non-contingent incentive (a $2 bill), which has been shown to increase participation rates [31]. Subjects receive $20 to complete each evaluation. Evaluation includes a series of questions about satisfaction with services received, current smoking status, and the history of quitting since enrolling in the study. Evaluators also ask about confidence in quitting or staying quit, presence of other smokers in the home, and use of other behavioral counseling services or quitting aids (nicotine patches or other nicotine replacement therapy, Zyban or Chantix).

Our study is participating in the CHART biochemical verification study as described in Riley *et al.*, which aims to obtain an estimate of the validity of self-report across consortium projects [28]. Subjects who indicate they are not smoking at the six-month evaluation are offered $100 to return a saliva sample, which is then tested for cotinine levels at Salimetrics laboratory in Pennsylvania [32]. In addition to providing data from these subjects to the consortium’s verification study, we have embedded a randomized design into the cotinine procedure that will test the impact of the amount of incentive offered and the smoking status on the rate of compliance with the saliva sample request. Stratifying by smoking status at the six-month evaluation, all self-reported nonsmokers and a random selection (25%) of self-reported smokers will be randomly assigned to be offered $20 or $100 for the return of the saliva sample. The results from the self-reported nonsmokers who were offered $100 will be included in the CHART biochemical verification study. The remaining conditions will allow us to determine whether smoking status and size of the incentive offered affect the return rates, information that can inform subsequent work in the area of hospitalized smokers.

At the two- and six-month evaluations, subjects are asked about whether they have been re-admitted to a hospital since their enrollment in the study. Information is obtained about length of each stay and reason for admission. In addition, research staff will perform a chart review of hospital records for enrolled subjects to determine re-hospitalization rates. If the chart review indicates relatively high accuracy of self-report, analysis of re-admission will use self-report data.

### Process measures

Every opportunity is taken to collect data about the implementation and fidelity of the various interventions. The number of smokers assessed and provided with brief counseling is obtained from hospital records. We track whether nicotine patches were distributed as planned or whether the patches were sent after discharge. The number and timing of proactive calls made to engage subjects in counseling is tracked in the quitline database. Other information tracked includes subjects’ participation in quitline counseling and the number of counseling calls provided. The quitline also routinely tracks the content of the counseling calls, and the length and timing of calls relative to the quit date, a process that is monitored for this study as well.

### Analytic plan

#### Primary aims

The primary aim of this study is to determine the effect of proactive telephone counseling and nicotine patch use on successful quitting among hospitalized smokers. The unit of analysis is the individual, but we anticipate some clustering due to commonalities in subjects admitted to individual hospitals (and, correspondingly, variability between hospital populations). generalized linear (binomial) mixed models are used to study the effect of treatment and clustering is accounted for with hospital-specific random effects [33]. The primary outcome is self-reported thirty-day abstinence at six months. The primary analysis is intent-to-treat and focuses on assessment of the main effects associated with proactive quitline counseling and receipt of nicotine patches at discharge. Analyses will be performed using SAS software (version 9.2, SAS Institute, Cary, NC). Although the study was not powered to detect an interaction, the interaction between the two treatments is assessed as a secondary outcome, as is seven-day abstinence, and the disagreement between cotinine-validated abstinence and seven-day self-reported abstinence. We will use Hochberg’s [22] step-up procedure to ensure a family-wise error rate/composite Type I error of ≤0.05.

#### Missing and incomplete data

At each step there is likely to be significant loss-to-follow-up in this population. The standard approach to imputation in cessation studies is to assume that any subject unavailable for evaluation has relapsed, although this approach has limitations [34]. The generalized linear mixed model is robust only to certain kinds of missing data [35]. Subjects who have relapsed may be more or less likely to submit to evaluation. Therefore, we will use subject-matter expertise and baseline characteristics to compile a range of plausible models for nonrandom nonresponse and we will carry out a sensitivity analysis over this class of models [36,37]. In addition, this allows us to examine whether inferences based on the complete-case sample are robust to these forms of nonrandom nonresponse or determine the circumstances under which said inferences would fail to hold [38]. Subjects who have not relapsed at the time of their last evaluation contact are censored. Because it is unlikely that censoring is independent of treatment assignment (for example, less censoring in the usual care), sensitivity analysis of all survival aims will be conducted as described above. We will model several possible mechanisms for informative censoring and determine the extent to which inferences are robust to the various assumptions [39,40].

#### Secondary aims

Secondary aims include assessing the possible moderating effects of diagnosis and determining the cost-effectiveness of the interventions. We will examine if subjects’ diagnosis (cardiovascular-pulmonary versus other diagnosis) moderates the effects of the intervention.

Analysis of cost-effectiveness depends on establishing an analytic perspective. In this study, we assume the hospital and quitline have the goal of maximizing benefits for subjects. We analyze cost per quitter. We assume that general costs are distributed evenly across callers since smokers are randomized. Personnel costs vary by position (for example, students have lower salaries than RTs) so staff time uses salary and benefits based on the personnel completing the various tasks (mailing materials, completing baseline assessment, and so on). We examine smokers who quit and re-hospitalizations prevented to establish the cost-effectiveness ratios for each intervention, in addition to (relative) quit and abstinence rates. To assess cost-effectiveness from the patient perspective, we evaluate quality-adjusted life years (QALYs). Quality of life weights are based on the health literature and life expectancy or, when necessary, modeled from clinician judgment of potential disease courses [41].

### Quality assurance

Quality assurance has been critical to the success of the studies conducted by our research team at CSH. Using the procedure developed during a pilot program, standardized training is conducted at all hospital sites to include a two-hour orientation to the project and procedural issues for all respiratory therapists (RTs) and recruitment staff, an independent Collaborative Institutional Training Initiative (CITI) training for UCSD staff consenting subjects (the modified two-hour NIH version is used for Scripps staff as per their IRB), a half-hour training for physicians to orient them to the program, a one-hour pharmacy orientation as well as a one-hour training of the quitline counselors. Standardized training ensures that all staff are trained to deliver the intervention to study subjects with retraining as needed. The implementation of the intervention is evaluated and monitored on an individual basis both during and after the training process. To confirm that providers deliver the same intervention at the different sites, we have monthly meetings with hospital staff. This study uses experienced quitline counselors. Counselors meet weekly in their supervision groups to discuss programmatic and clinical issues.

## Discussion

This effectiveness trial employs a 2 x 2 factorial design to tease out the effects of two cessation interventions for hospitalized smokers. The randomized design is used here because strong real-world evidence for the effectiveness of interventions with hospitalized smokers is still needed. A 2007 Cochrane Review on this topic has found that for counseling to be effective with hospitalized smokers it needs to extend to at least one month post-discharge [6]. Counseling of this length is common for efficacy trials, but it is rare in real-world operations; hospital staff cannot typically follow up patients this long for preventive care. Moreover, the Cochrane Review found that nicotine replacement therapy (NRT), which has been shown efficacious in many trials [16], has little effect with hospitalized smokers [6]. The causes for the lack of NRT effect in this context are poorly understood. For these reasons, we test the proposed interventions, which include both dispensing nicotine patches and providing proactive telephone counseling, in a randomized factorial design.

The innovation of the study is in its ability to address several key issues in the field, all in one design:

1. It will answer the question of whether giving nicotine patches to smokers at discharge will increase the long-term quit rate, compared to what is naturally happening already. Since some hospitals may not be ready to link with a quitline, the study design will provide information as to whether the option of providing nicotine patches is sufficient to generate a statistically meaningful difference.

2. It will answer the question of whether linking hospital staff counseling with quitline counseling can increase the quit rate. The study will also provide detailed operational information on how much effort the quitline and the hospital have to put in to execute a counseling program with hospitalized smokers.

3. It will find out if providing both patches and counseling will be better than either intervention alone for this population. Since the costs of providing patches and counseling are relative easy to calculate in this case, this will allow a comparison on cost-effectiveness of these different interventions.

At the same time, the study is building a working model of linking hospital staff work flow with an existing state quitline operation, which may be important for hospitals that adopt tobacco cessation with its required post-discharge follow-up as one of the accreditation performance standards.

Telephone counseling is one of the few empirically validated behavioral services that has become institutionalized. Smokers can access quitline services from anywhere in the U.S. As a result, this study may provide a successful and sustainable model linking hospitals to existing state quitline operations. The ubiquity of quitlines and the consistency of counseling delivery through the centralized state operation make the quitline-hospital partnership attractive. The costs of continued smoking by hospitalized patients are high, both to the individual and to society. Providing proactive smoking cessation counseling or nicotine patches to hospital patients is expected to reduce the risk of relapse and re-hospitalization. The poor health outcomes and high re-hospitalization rates associated with continued smoking make a compelling case for an effective and cost-effective public health model such as that represented by the quitline-hospital partnership.

## Trial status

This study has been approved by the relevant IRBs and recruitment is ongoing.

## Abbreviations

CHART : Consortium of Hospitals to Advance Research on Tobacco; CITI : Collaborative Institutional Training Initiative; CSH : California Smokers’ Helpline; DSMB : Data and Safety Monitoring Board; EMR : Electronic medical record; EPHI : Electronic protected health information; HIPAA :Health Insurance Portability and Accountability Act; HRPP : Human Research Protections Program; IRB :Internal Review Board; NIH : National Institutes of Health; NRT : Nicotine replacement therapy; QALY : Quality-adjusted life years; RR : Research recruiter; RT : Respiratory therapist; UC : Usual care; UCSD : University of California San Diego.

## Competing interests

The authors declare that they have no competing interests.

## Authors’ contributions

All authors agreed on the need for the paper. SHZ conceived of the study and the design. SC, CK and HKC drafted the paper. AG assisted with design issues and was responsible for the data analysis plan. KB conducted pilot work and helped with implementation issues. EC at Scripps and TM, GS, and JL at UCSD provided leadership for the hospitals to participate in the study. All authors read and approved the final manuscript.
